# A case report of tumor-associated liver injury alleviated with antineoplastic drug

**DOI:** 10.3389/fonc.2025.1644790

**Published:** 2025-11-20

**Authors:** ZiRu Yang, Lixia Cao, Wentao Kuai, Shengjuan Yao, Shuang Li, Liang Xu

**Affiliations:** 1 Clinical School of the Second People’s Hospital, Tianjin Medical University, Tianjin, China; 2 Tianjin Institute of Hepatology, Tianjin Second People’s Hospital, Tianjin, China; 3 Department of Infectious Diseases, JiZhou District People's Hospital of Tianjin, Tianjin, China

**Keywords:** tumor, metastases, liver injury, anti-tumor, therapy

## Abstract

Tumors are the major diseases that threaten people’s health and life at present. Liver injury often happens in patients with cancer, and which mostly attribute to application of anti-cancer drugs. However, some cases caused by the infiltration and destruction of metastatic cancer, and anti-tumor drugs play a key role in restoring liver function instead of inducing drug induced liver injury (DILI). This article reports a case of tumor-associated Liver Injury (TALI) who suffered HER2-negative metastatic breast cancer with liver metastasis, but her liver function was recovery after treatment with Alpelisib, an anti-tumor drug, as meanwhile as achieving anti-tumor. Through this case we aims to emphasize to differentiate the causes of TALI and make the treatment strategies accordingly.

## 1 Case presentation

**Figure 1:**
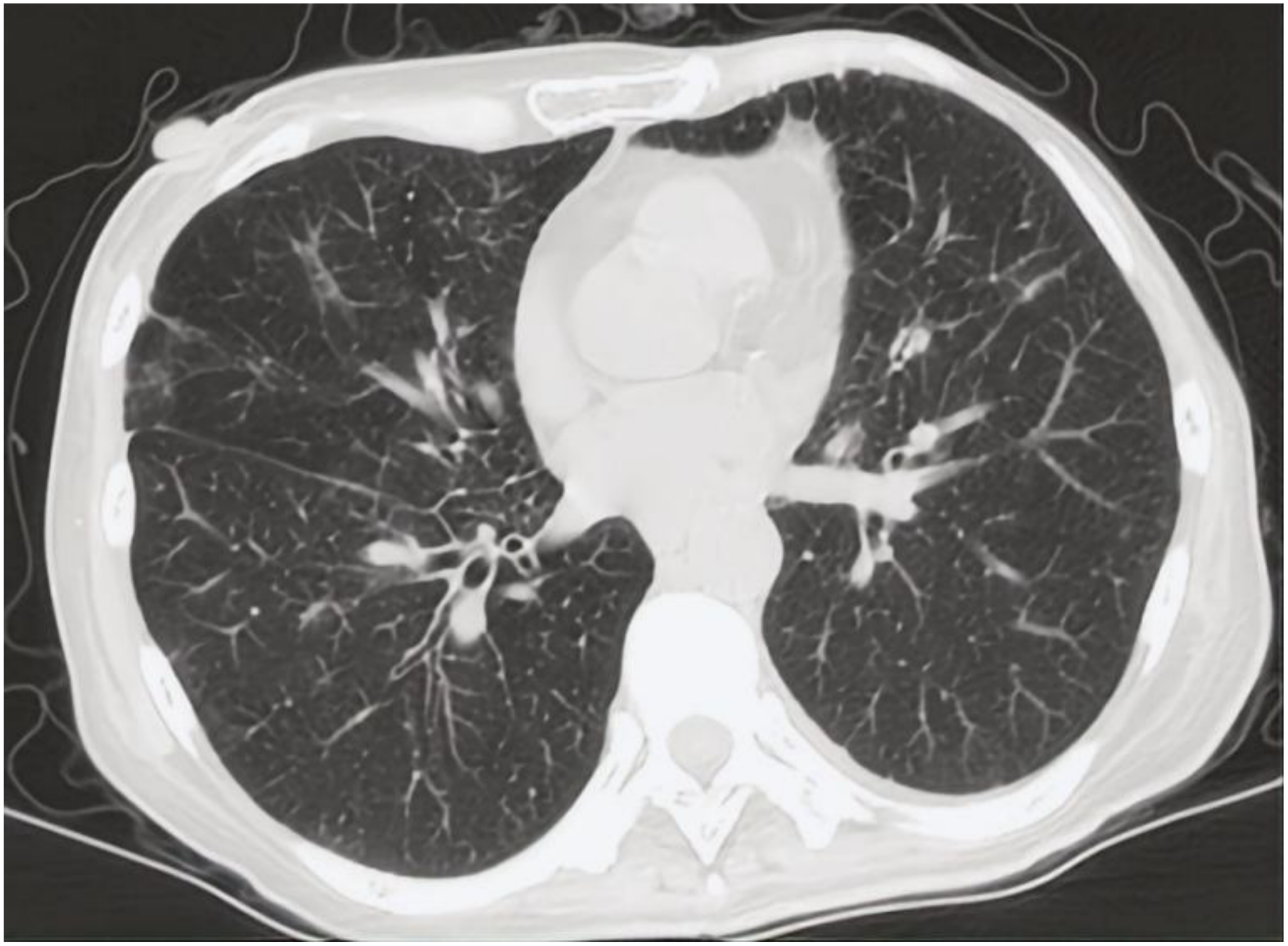
CT scan of chest showed multiple small nodules considered as metastatic carcinoma in both lungs.

In 2024, a 45kg female patient who was admitted due to fatigue, anorexia, and dark yellow urine for 4 months. Her abdominal distension occurred 4 months ago, and then developed fatigue, loss of appetite, dark yellow urine, and mild abnormal liver function, but she did not take any medical treatment, and these symptoms became worsen in 5 days. 6 years before, she was diagnosed with breast cancer and received surgically resected, multiple rounds of radiotherapy, chemotherapy, and targeted therapy. 4 months before, she was monitored with recurrence of breast cancer and distant metastasis to brain and bones, and begun cranial radiotherapy, meanwhile, the systemic therapies, including targeted therapy, were discontinued. The patient concurrently begun a regimen of anti-tumor Chinese herbal decoctions. She has no family history of liver disease. Physical examination: we found her with severe jaundice of the skin and mucous membranes, and liver palms, and the swelling liver exceeded the right xiphoid 3cm, with a hard texture. Laboratory tests: autoimmune antibodies and serum markers of hepatitis virus were negative. Chest computerized tomography (CT) scan showed multiple small nodules in both lungs, local atelectasis in the right middle lobe and left lower lobe, and postoperative changes were consistent with the loss of the left breast (**Figure 1**). Suspicious nodules can be seen in some vertebrae, which require further examination. Abdominal CT showed multiple low-density lesions in the liver, ascites, and accessory spleen. MRCP showed that the bile duct in the liver portal area was unclear, which may be due to tumor invasion or external compression, accompanied by uneven expansion of the upper intrahepatic bile duct. (**Figure 3**).

**Figure 2:**
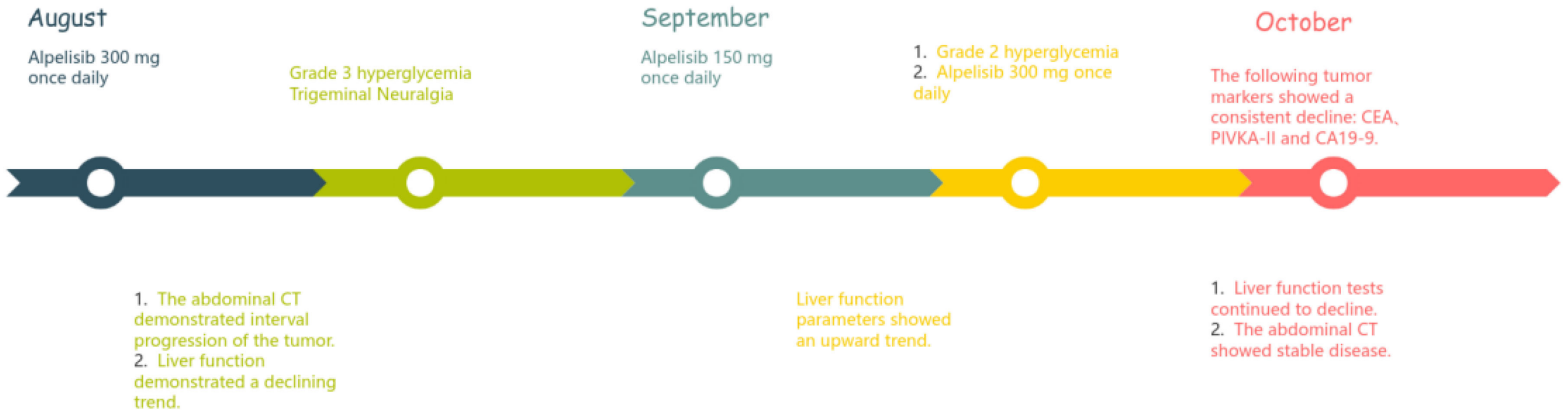
Timeline showing treatment and health updates from August to October.

**Figure 3:**
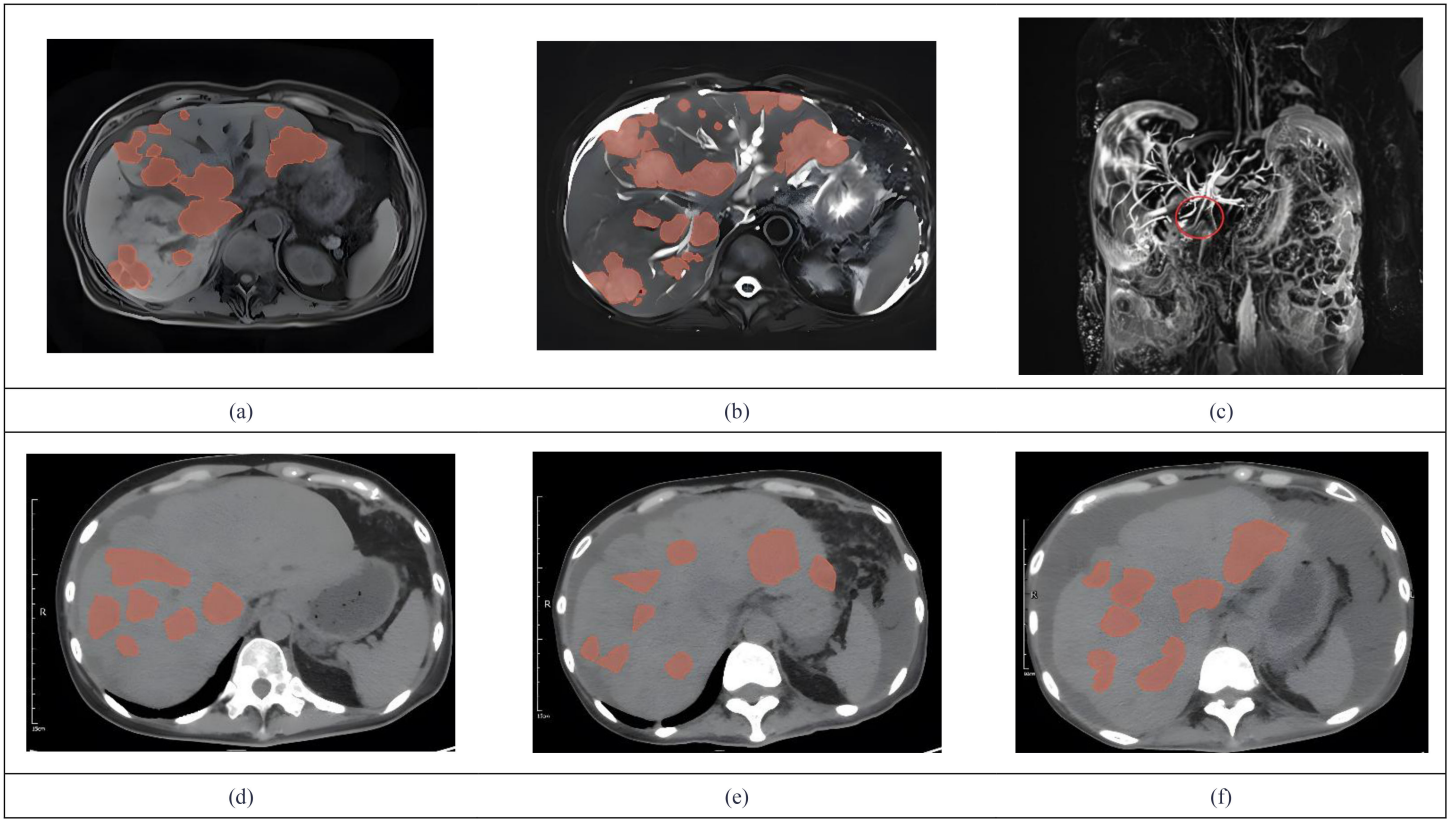
We used 3D Slicer (Version 5.9.0) to manually delineate the lesions by employing the “Draw” or “Paint” tools within the “Segment Editor” module. MRCP showed tumor invasion **(a)** Metastases demonstrate hypointensity on T1WI. **(b)** Metastases demonstrate hyperintensity on T2WI. **(c)** The coronal MRCP image showed a biliary duct interruption at the marked site. **(d)** The abdominal CT in July showed multiple low-density lesions in the liver. **(e)** Abdominal CT in august revealed progression of multiple low-density liver lesions both in number and size. **(f)** Abdominal CT in October showed no significant progression of tumor lesions.

A diagnosis was considered as follows: 1.Drug induced liver injury (DILI) 2. Secondary malignancy in the liver; 3. Distant metastasis of breast cancer 4. Ascites.

**Figure 4:**
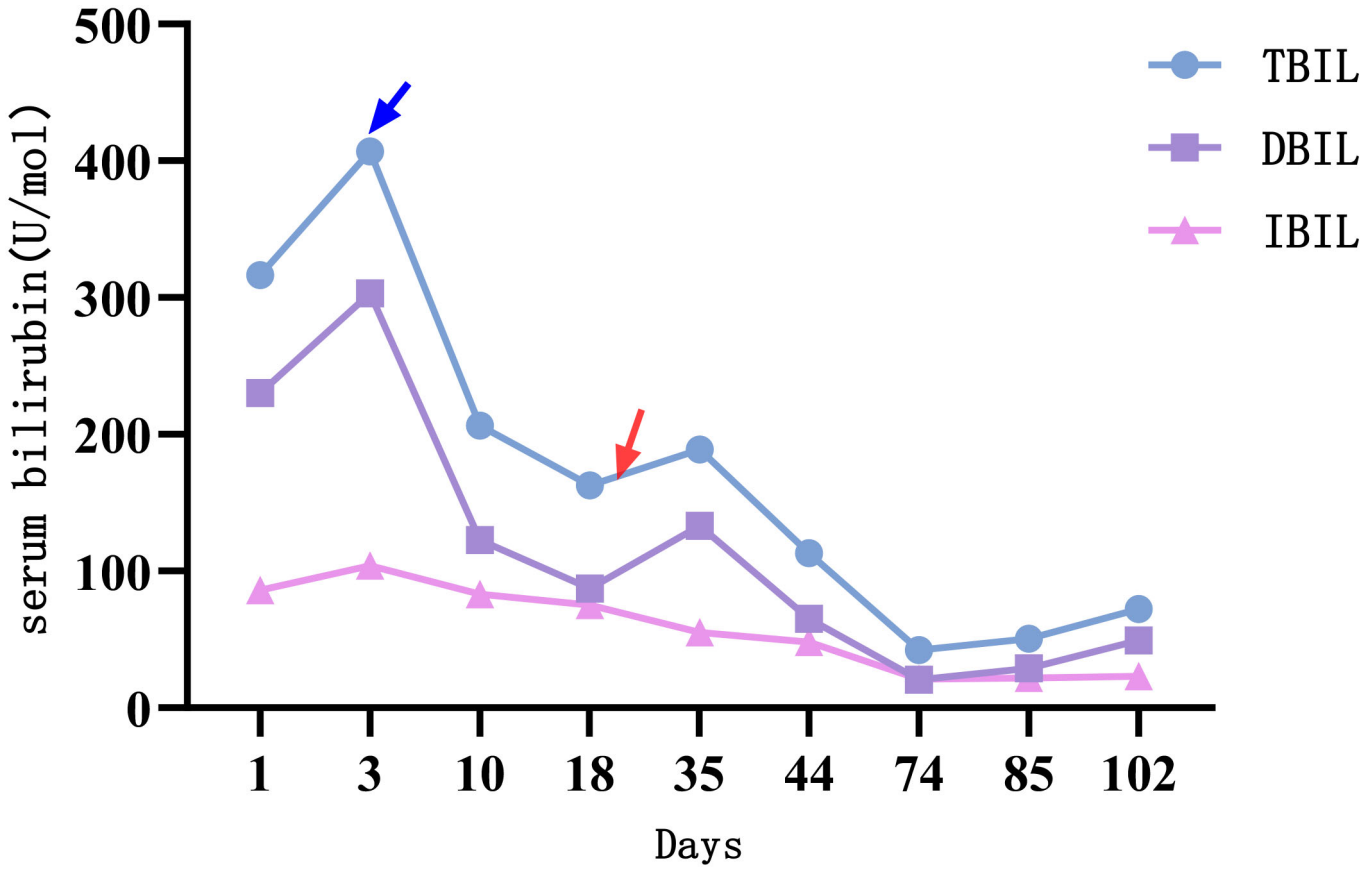
The changes of serum bilirubin indicators. Treatment with alpelisib 300 mg once daily was initiated at the time point indicated by the blue arrow. Subsequently, a significant decline was observed across all parameters of liver function, particularly serum bilirubin. At the time point marked by the red arrow, the dose of alpelisib was reduced to 150 mg once daily due to Grade 3 hyperglycemia and neuralgia. Following this dose reduction, serum bilirubin levels exhibited a transient elevation.

**Figure 5:**
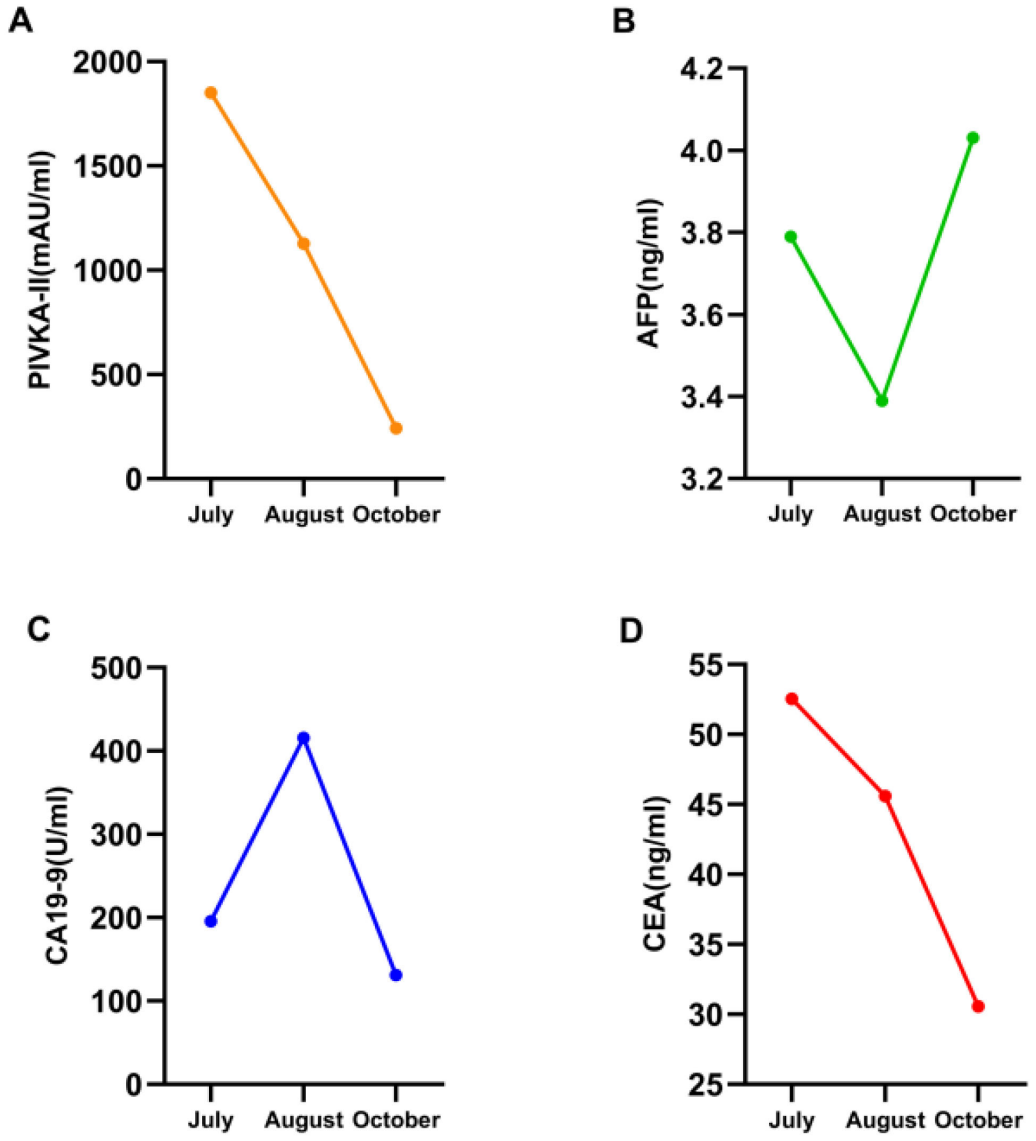
Four line graphs labeled **A** to **D**, showing changes in various biomarkers from July to October. Graph **A** shows a decrease in PIVKA-II levels. **(B)** AFP consistently remained normal. Graph **C** depicts an increase and then decrease in CA19-9 levels. Graph **D** shows a decline in CEA levels.

After admission, the patient was given a comprehensive treatment including oral bicyclol, magnesium isoglycyrrhizinate, adenosine butylene sulfonate, furosemide, spironolactone, liver protection therapy, diuretics and symptomatic treatment. Ascites was punctured and drained, and the ascites examination showed the characteristics of exudative fluid. Antibiotic therapy was applied, and intermittent albumin infusion and enteral nutrition support were given. Electrolyte analysis showed hyponatremia, and then tolvaptan was prescribed. But the liver function continued to deteriorate rapidly. The patient had ascites and refuses to undergone liver biopsy. After careful analysis and evaluation, the cause of liver injury was considered as tumor-infiltrating liver injury (TILI). Subsequently, a treatment of alpelisib at 300 mg once daily was initiated in early August to control tumor progression, and then the liver function indicators and tumor markers (PIVKA-II, CA19-9, and CEA) decreased significantly (see **Figures 4**, **5**) when liver metastases controlled. Changes in serological parameters are presented in **Table 1**.

**Table 1:** Trends in relevant metrics from July 24 through November 7.

Date	Jul 25	Aug 4	Aug12	Aug29	Sep 7	Oct 7	Oct 10	Nov 7	Ref.Range	Units
ALT	110.6	59.7	36.7	45.1	43.7	60.3	15.5	36	7-40	U/L
AST	306.6	306.6	164.7	312.7	312.7	129.3	60.3	109.5	13-35	U/L
TBIL	316.3	206.4	162.5	189.1	113.3	42.1	50.6	72.4	5.64-18.52	umol/L
DBIL	230.3	122.5	87.2	133.3	65.2	20.8	26.1	49.2	1.82-6.78	umol/L
Glu	5.18	23.22	11.4	4.23	4.82	6.39	16.08	5.16	3.9-6.1	mmol/l

The first side effect was refractory hyperglycemia, with the highest value of 32.73mmol/L (Fast blood glucose changes are shown in Appendix 1). Acarbose, dapagliflozin, metformin, and insulin were prescribed to lower blood glucose. Fast blood glucose levels were maintained between 9 and 14 mmol/l, consistent with alpelisib-induced Grade 2 hyperglycemia. The second side effect was neuralgia. In response to facial neuralgia and numbness in the hands and feet that developed in early September, the patient was prescribed acetaminophen, oxycodone, bucinazine for pain relief, and Vitamin B complex for nerve support. Due to inadequately controlled facial neuralgia and the development of Grade 3 hyperglycemia, the dose of alpelisib was reduced by half to 150 mg once daily in September. Liver function indicators, such as bilirubin increased soon after the anti-tumor drug alpelisib halved in dose, conversely, they recovered immediately as the dose of alpelisib added. Throughout this interval, glycemic management was intensified and the original dosage of alpelisib was resumed. The timeline is depicted in **Figure 2**.

## 2 Discussion

**Figure 6:**
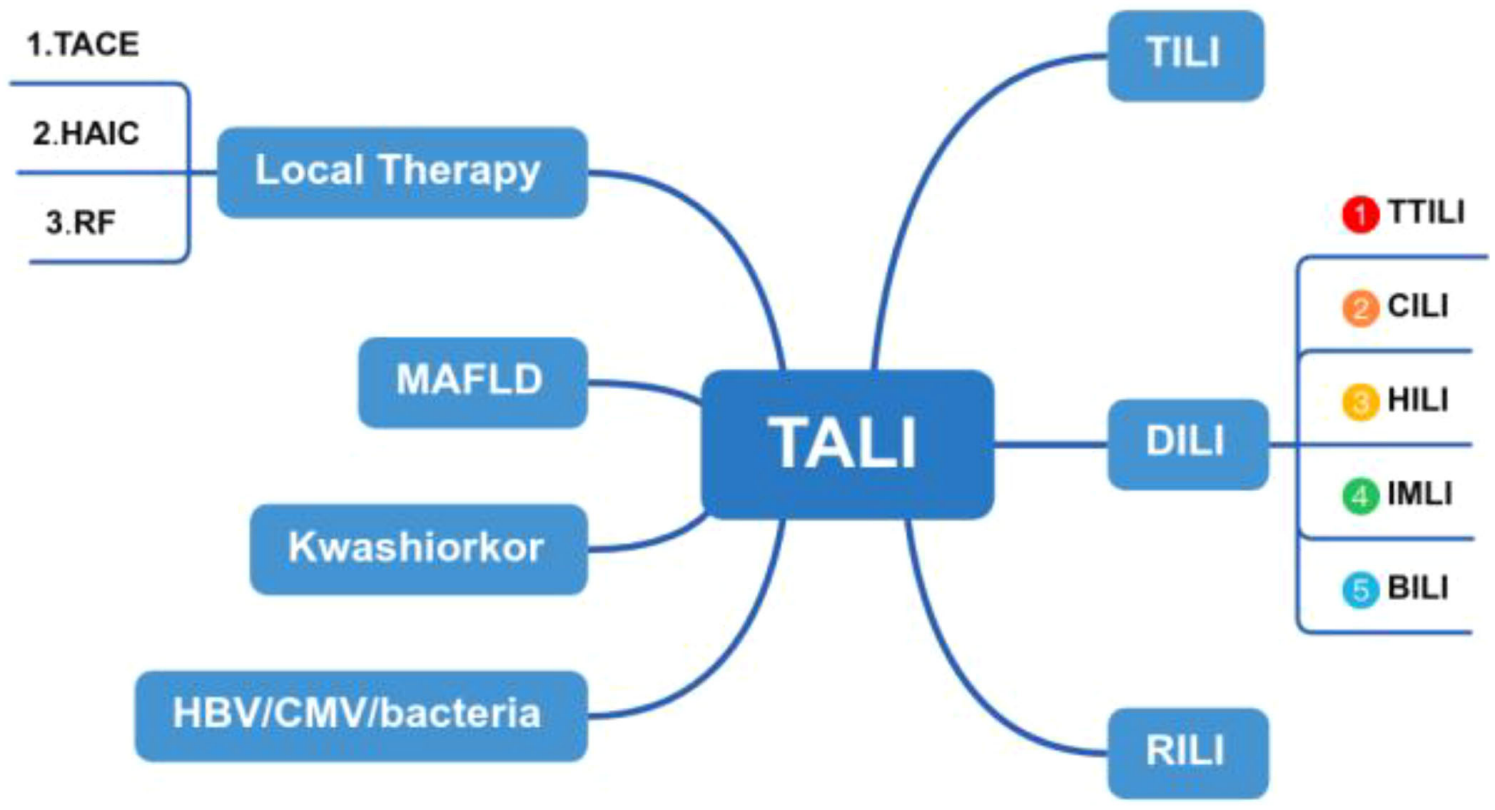
Flowchart illustrating the components of TALI. TALI, Tumor-associated liver injury; TILI, Tumor-infiltrating liver injury; RILI, Radiation-induced liver injury; DILI, Drug-induced liver injury; TTILI, Targeted Therapy-induced Liver injury; CILI, Chemotherapy-induced liver injury; HILI, Herb-induced; liver injury, IMLI, Immune-mediated liver injury; BILI, Biologic-induced liver injury.

Studies have shown that the incidence of tumor-associated liver injury is affected by tumor type, treatment, and primary liver disease in patients with liver cancer. The main causes of liver injury include chemotherapy, targeted therapy, immunotherapy, biologic therapy, and various local therapies et al, (**Figure 6**) which can be divided into the following types: (1) Drug-induced liver injury: chemotherapy drugs, targeted therapy drugs and biologic Therapy cause liver injury (1). (2) Local therapy, such as Radiation-induced liver injury: The liver is an important target organ for radiotherapy. When exposed to radiation, it can cause acute radiation hepatitis, chronic fibrosis, and even cirrhosis (2). (3) Immune-mediated liver injury: Immune checkpoint inhibitors (ICIs), as an emerging tumor treatment method, have received increasing clinical attention because they can induce immune-related liver injury (3). (4) Tumor-infiltrating liver injury: Liver metastasis is another important cause of liver injury. The differential diagnosis of TALI for this patient with liver injury was broad. We should consider drug-induced liver injury and biliary obstruction as part of the differential diagnosis. Hepatocellular injury is typically characterized by elevated ALT or AST levels. In this context, ALT rises more rapidly than AST, and the extent of its increase is directly proportional to the scope of hepatocyte necrosis. In cases of mild injury, ALT does not exceed five times the upper limit of normal, whereas in severe injury, it can exceed ten times the upper limit. In contrast, cholestasis is primarily indicated by elevated ALP and GGT levels, often accompanied by an increase in bilirubin. ALP typically rises above two times the upper limit of normal, while GGT can reach five to ten times the upper limit (4). In non-contrast CT scans, hepatocellular injury-type damage may present as diffuse hepatomegaly, a homogeneous decrease in liver parenchyma density, and a reduced liver-to-spleen density ratio (normal ratio >1, which becomes <1 post-injury). On MRI T2-weighted images, a mild signal increase in the liver parenchyma can be observed, indicating the presence of hepatocellular edema. For cholestatic liver injury, MRCP can be utilized to reveal rigidity and rarefaction of the intrahepatic small bile ducts. In severe cases, segmental bile duct dilation may appear, although the common bile duct typically shows no significant changes (5). In this case, the diagnosis of infiltrative liver injury due to tumor has been established.

In this context, tumor-infiltrating liver damage was considered, with its underlying pathological mechanisms recognized as multifaceted and complex. One major aspect is tumor cell infiltration, where tumor cells disseminate to the liver via the bloodstream or lymphatic system and proliferate within hepatic tissue. This invasion disrupts liver architecture through the secretion of cytokines, matrix metalloproteinases (MMPs), and other molecular mediators, ultimately impairing liver function (6). Hepatic metastases commonly originate from colorectal, gastric and breast cancers, with tumor cells colonizing the liver, forming metastatic lesions that progressively degrade hepatic parenchyma and induce hepatocyte damage.

Another significant factor is immune-mediated liver injury, which arises from interactions can trigger localized immune reactions that contribute to liver damage. Tumor-associated macrophages (TAMs) play a crucial role in tumor progression by secreting proinflammatory cytokines (e.g., IL-1, IL-6) and proteolytic enzymes, further exacerbating liver injury (7).

Hepatic hemodynamic alterations also contribute to liver damage. Tumor infiltration can obstruct hepatic blood flow, leading to regional ischemia, structural disorganization of hepatic lobules, hepatocyte necrosis, and worsening liver injury. Additionally, tumor cells stimulate the formation of new blood vessels through angiogenesis. However these neovessels often exhibit structural irregularities, making them prone to localized blood flow obstruction, further exacerbating liver dysfunction (8).

Finally, Liver fibrosis and cirrhosis may develop as a consequence of chronic inflammation induced by tumor infiltration. This process activates hepatic stellate cells, promoting deposition of extracellular matrix components such as collagen, which accelerates liver fibrosis and, if persistent, may lead to cirrhosis. Moreover, tumor cells evade immune surveillance by secreting immunosuppressive factors such as transforming growth factor-beta (TGF-β). This not only facilitates liver fibrosis but also impairs immune cells function, further compromising the liver^’^s ability to counteract tumor progression (9).

In this case, the effect of liver protection drug treatment was poor because of diffuse liver damage caused by tumor liver metastasis infiltration. In addition, the patient had a history of breast cancer, multiple rounds of radiotherapy, chemotherapy, and targeted therapy, and breast cancer recurred, resulting in multiple distant metastases, including brain and bone metastases, and a history of brain radiotherapy. Physical examination revealed jaundice, ascites, and hepatomegaly. After the administration of anti-tumor drugs, bilirubin improved significantly, but there are also intermittent adverse reactions. The use of anti-tumor drugs during this period help to improve liver function indicators. Alpelisib is an oral PI3Kα-specific inhibitor that plays a key role in regulating cell proliferation, differentiation, and survival. In an open-label, single-group, phase 1b clinical trial (NCT01219699), the most common serious adverse events (SAEs) were hyperglycemia (22%) and maculopapular rash (13%), which were attributed to impaired glucose uptake and gluconeogenesis associated with PI3K inhibitors (10). The liver is one of primary target organs for breast cancer metastasis, as it receives dual blood supply from the main target organs for breast cancer metastasis because it receives dual blood supply from the portal vein and hepatic artery, which promotes disease progression. According to the “seed and soil” hypothesis, the liver microenvironment is the best “soil” for cancer cell proliferation. The “epithelial-mesenchymal transition (EMT)” theory believes that microRNAs plays a vital role in the regulatory mechanism. Inflammatory factors in the tumor microenvironment may enhance the invasiveness of tumor cells to the liver. During liver metastasis, metastatic tumor cells adhere to vascular endothelial cells, and TNF-α-induced endothelial E-selectin is a key factor. The loss of promoter methylation leads to the expression of E-cadherin by breast cancer cells. In addition, the liver microenvironment can induce breast cancer cells to re-express E-cadherin, restoring them to an epithelial phenotype, which contributes to the occurrence of mesenchymal-epithelial transition (MET) (11). The prognosis of breast cancer liver metastases is mainly affected by the molecular classification of metastatic lesion receptor changes is still unclear. Currently, popular hypotheses focus on intratumoral heterogeneity, clonal evolution, and treatment-induced selection pressure. However, not all patients with tumor infiltration show improved liver function after antitumor therapy. The applicability of anti-tumor medications varies by tumor infiltration characteristics, necessitating personalized oncologic therapeutic strategies.

## 3 Conclusion

In this clinical case of liver injury caused by infiltration and destruction of liver metastasis cancer, anti-tumor drugs showed effect of restoring liver function, thus providing an important method and direction for patients with TILI.

## Data Availability

The original contributions presented in the study are included in the article/supplementary material. Further inquiries can be directed to the corresponding author.
