# Supplementary material for: A case report of tumor-associated liver injury alleviated with antineoplastic drug
Source: Front Oncol. 2025 Nov 20;15:1644790. doi: 10.3389/fonc.2025.1644790 (PMC12675193; doi:10.3389/fonc.2025.1644790)
Supplement: Supplementary file 1 [file Image1.pdf]

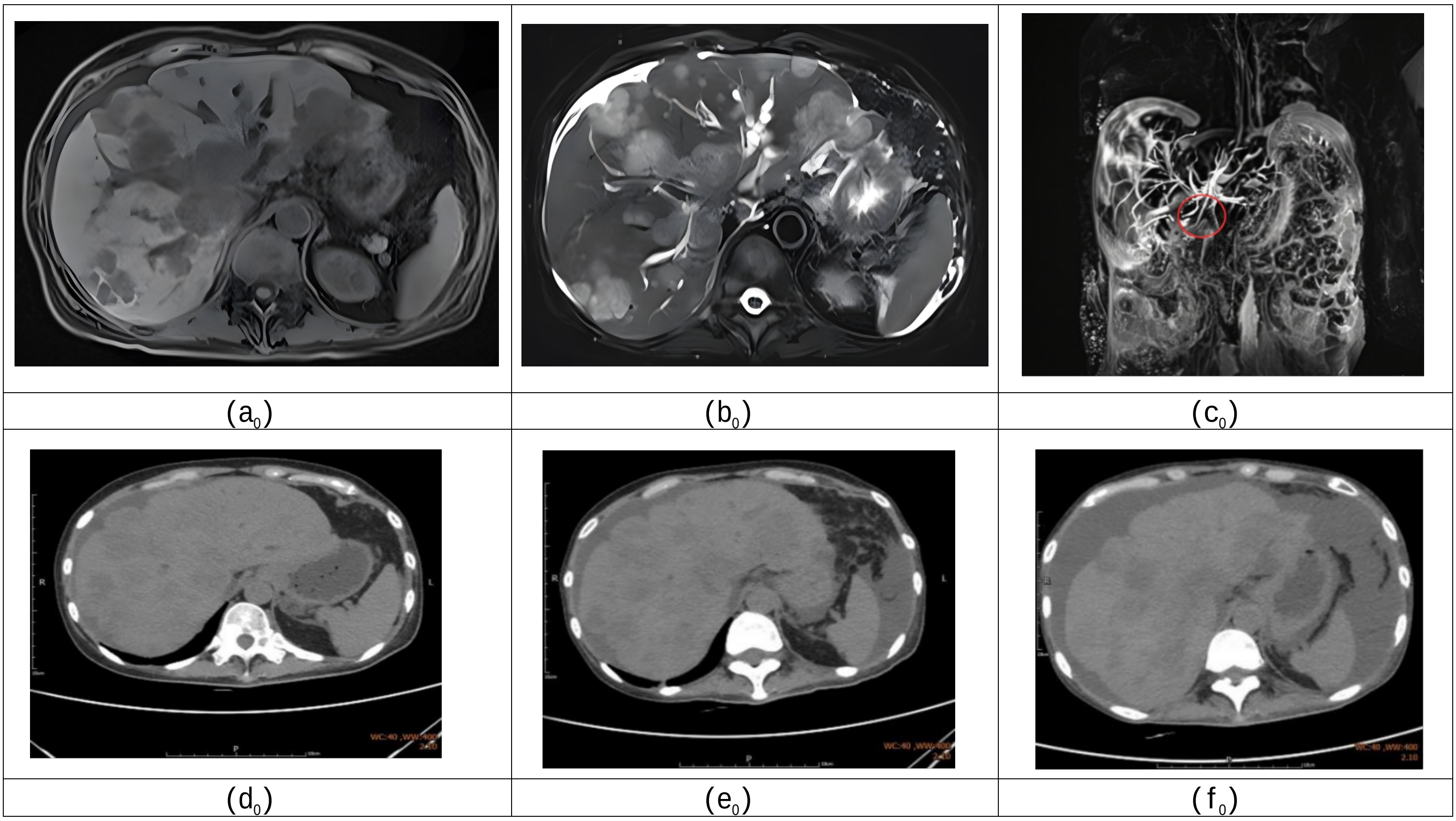

Supplementary 3. We used 3D Slicer (Version 5.9.0) to manually delineate the lesions by employing the "Draw" or "Paint" tools within the "Segment Editor" module. MRCP showed tumor invasion(a0)Metastases demonstrate hypointensity on T1WI. (b0)Metastases demonstrate hyperintensity on T2WI. (c0)The coronal MRCP image showed a biliary duct interruption at the marked site. (d0)The abdominal CT in July showed multiple low-density lesions in the liver.(e0) Abdominal CT in august revealed progression of multiple low-density liver lesions both in number and size.(f0) Abdominal CT in October showed no significant progression of tumor lesions.
