# Supplementary material for: A case report of tumor-associated liver injury alleviated with antineoplastic drug
Source: Front Oncol. 2025 Nov 20;15:1644790. doi: 10.3389/fonc.2025.1644790 (PMC12675193; doi:10.3389/fonc.2025.1644790)
Supplement: Supplementary file 3 [file DataSheet2.pdf]

Lab Test/Ref.Range/U	25-Jul-2024	4-Aug-2024	12-Aug-2024	29-Aug-2024
ALT(7-40)U/L	110.6	59.7	36.7	45.1
AST(13-35)U/L	306.6	306.6	164.7	312.7
GGT（10-60）U/L	323.8	622.6	103.3	1371.6
ALP（45-125）U/L	685.5	787.1	878.9	1184.3
ALB（40-55）g/l	26.1	35.7	37.3	26.2
TBIL(5.64-18.52)umol	316.3	206.4	162.5	189.1
DBIL(1.82-6.78)umol/	230.3	122.5	87.2	133.3
GLU(3.9-6.1)mmol/l	5.18	23.22	11.4	4.23
PIVKA-II(0-40)AU/ml	1850			1128
CA-19-9(0-35)U/ml	195.61			415.67
CEA(0-5)ng/ml	52.53			45.59
AFP(0-10)ng/ml	3.79			3.39
Ascites(cm)	5		6.5	

7-Sep-2024	7-Oct-2024	10-Oct-2024	7-Nov-2024
43.7	60.3	15.5	36
312.7	129.3	60.3	109.5
1386.1	1015.6	1497.3	1400
919.8	709.5	968.6	1241.2
29.8	31.7	32.1	26.8
113.3	42.1	50.6	72.4
65.2	20.8	26.1	49.2
4.82	6.39	16.08	5.16
	243		
	130.89		
	30.55		
	4.03		
5.5		8.8	8.3
